# Supplementary material for: Using Dynamic Bayesian Optimization to Induce Desired Effects in the Presence of Motor Learning: a Simulation Study
Source: bioRxiv. 2024 Aug 16:2024.08.13.607783. Preprint. [Version 1] doi: 10.1101/2024.08.13.607783 (PMC11343104; doi:10.1101/2024.08.13.607783)
Supplement: Supplement 1 [file NIHPP2024.08.13.607783v1-supplement-1.pdf]

bioRxiv preprint doi: https://doi.org/10.1101/2024.08.13.607783; this version posted August 16, 2024. The copyright holder for this preprint (which was not certified by peer review) is the author/funder, who has granted bioRxiv a license to display the preprint in perpetuity. It is made available under aCC-BY 4.0 International license.

7. Supplementary Materials

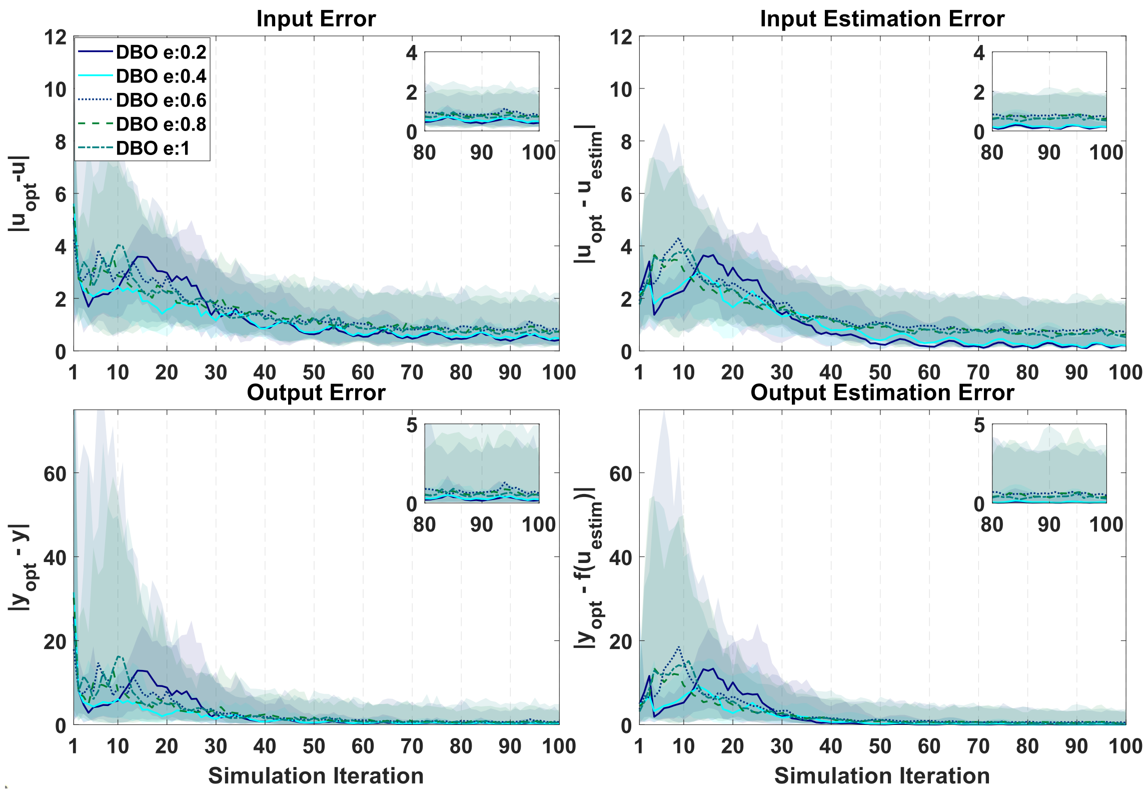

Figure S1: DBO optimizer performance on the selected model with explicit time dependence. The x-axis indicates the number of iterations during optimization (i.e., after three random inputs are initially applied). In all plots, solid lines indicate median and shaded regions of same color extend from the 20th to the 80th percentile across 100 repetitions at each iteration. Optimizer performance is quantified based on *input error* (top left), *input estimation error* (top right), *output error* (bottom left), and *output estimation error* (bottom right). An zoomed-in insert is provided in each panel to help visualize differences in the last 20 iterations.

bioRxiv preprint doi: https://doi.org/10.1101/2024.08.13.607783; this version posted August 16, 2024. The copyright holder for this preprint (which was not certified by peer review) is the author/funder, who has granted bioRxiv a license to display the preprint in perpetuity. It is made available under aCC-BY 4.0 International license.

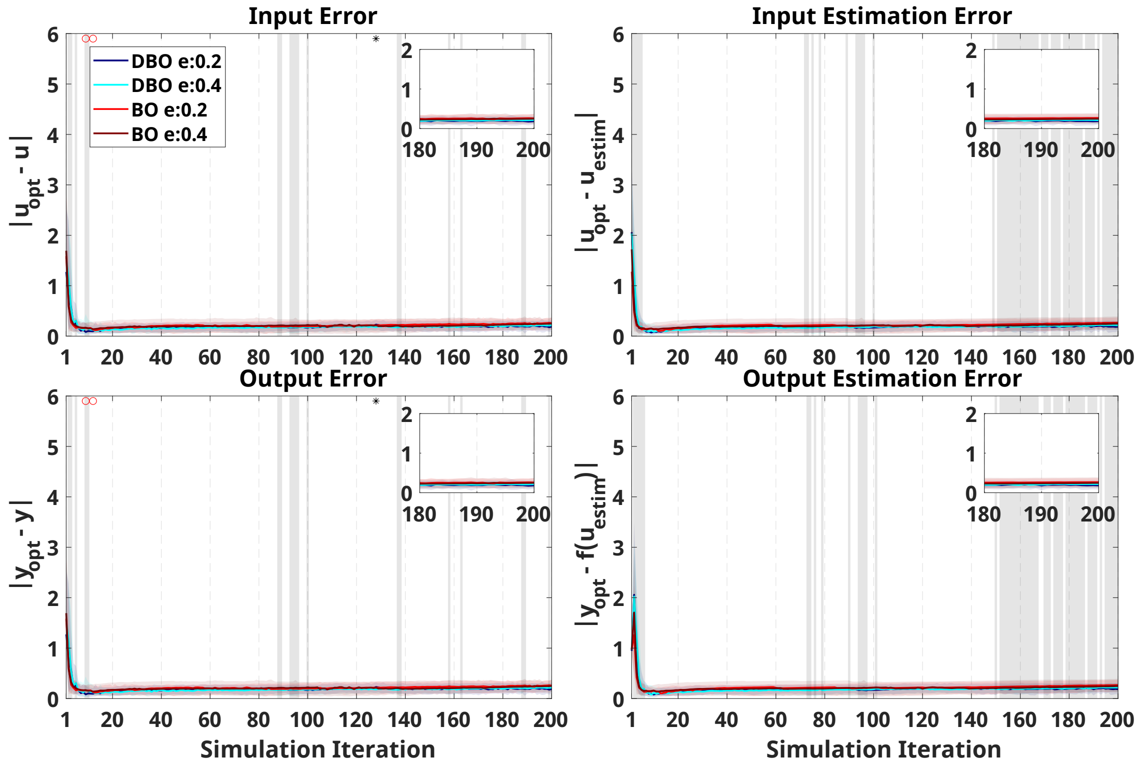

Figure S2: Virtual HIL simulation results using UDL model with slow negative learning (Model 1) as the virtual human response. The x-axis indicates the number of iterations during optimization (i.e., after three random inputs are initially applied). In all plots, the blue/cyan and red/crimson indicate simulation results using DBO with e-ratio of 0.2/0.4, and BO with e-ratio of 0.2/0.4, respectively. Solid lines indicate median and shaded regions of same color extend from the 20th to the 80th percentile across 50 repetitions at each iteration. Optimizer performance is quantified based on *input error* (top left), *input estimation error* (top right), *output error* (bottom left), and *output estimation error* (bottom right). An zoomed-in insert is provided in each panel to help visualize differences in the last 20 iterations. The gray shaded region indicates the presence of significant differences ($p_{unc} < 0.05$) between simulation results using different optimizers at each iteration. Asterisks at the top of each figure indicate a significant effect of the e-ratio, and red circles indicate a significant interaction between e-ratio and the optimizer on the outcome at each iteration.

bioRxiv preprint doi: https://doi.org/10.1101/2024.08.13.607783; this version posted August 16, 2024. The copyright holder for this preprint (which was not certified by peer review) is the author/funder, who has granted bioRxiv a license to display the preprint in perpetuity. It is made available under aCC-BY 4.0 International license.

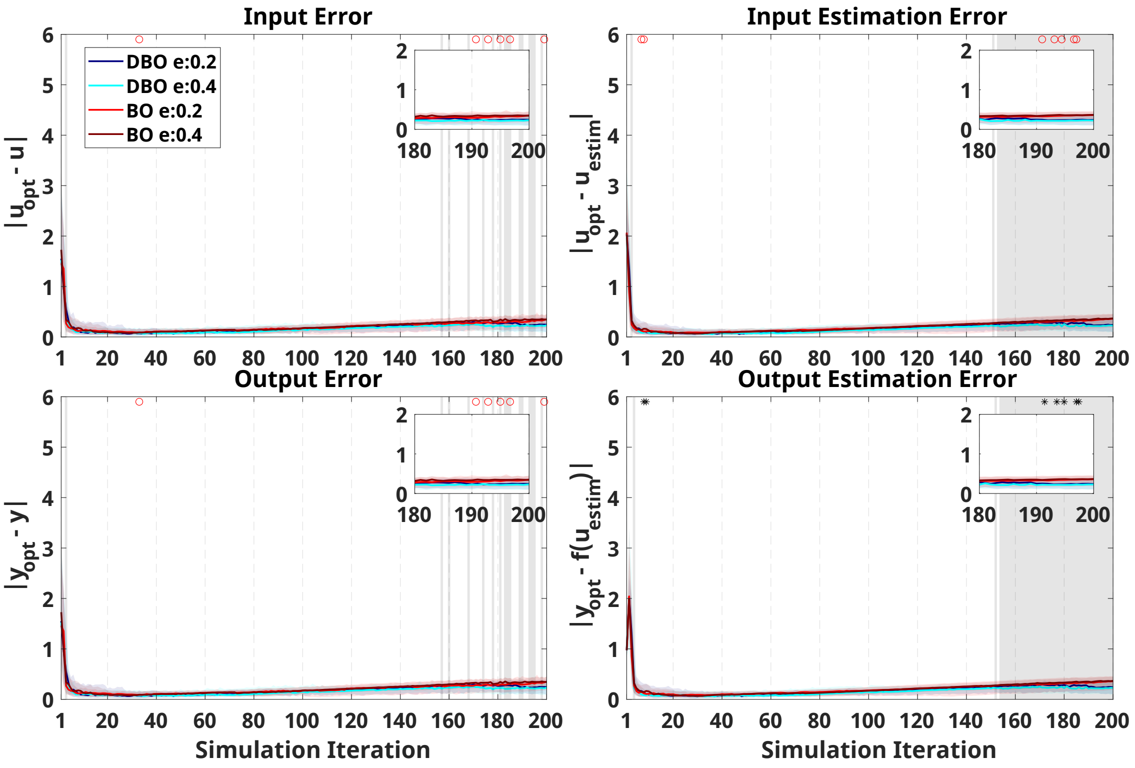

Figure S3: Virtual HIL simulation results using UDL model with slow positive learning (Model 2) as the virtual human response.

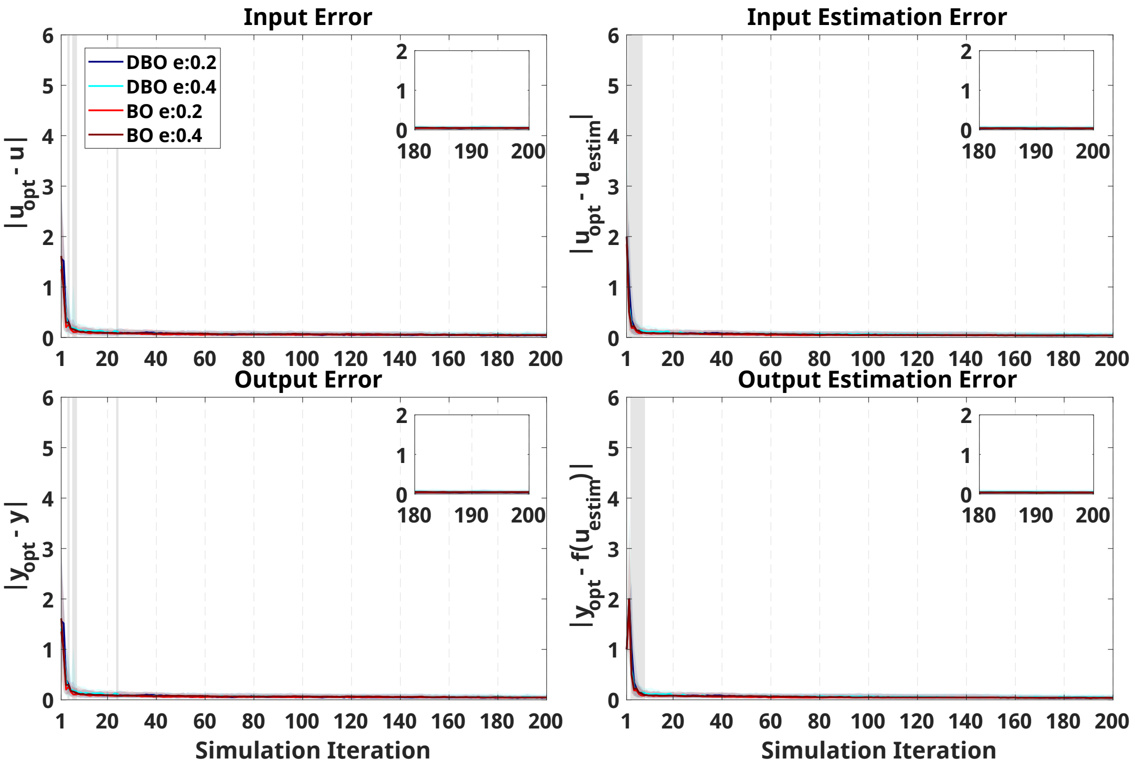

Figure S4: Virtual HIL simulation results using UDL model with constant and no learning (Model 5) as the virtual human response.

bioRxiv preprint doi: https://doi.org/10.1101/2024.08.13.607783; this version posted August 16, 2024. The copyright holder for this preprint (which was not certified by peer review) is the author/funder, who has granted bioRxiv a license to display the preprint in perpetuity. It is made available under aCC-BY 4.0 International license.

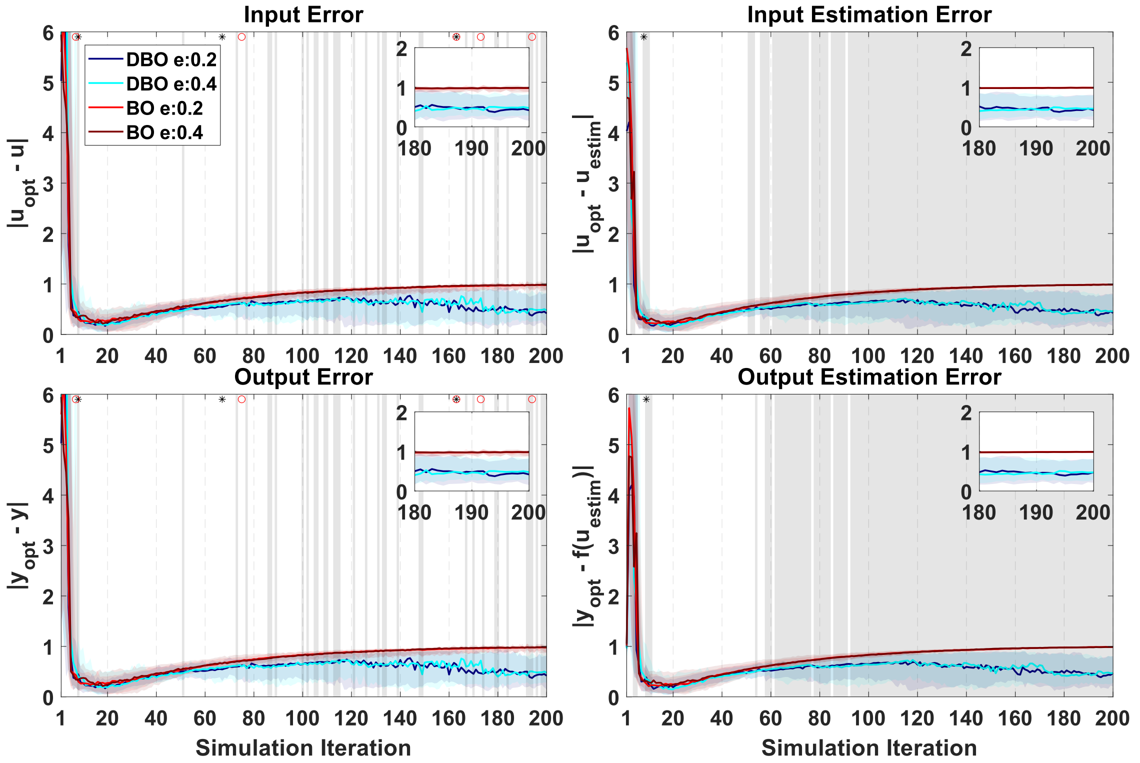

Figure S5: Virtual HIL simulation results using UDL model with only adaptation (Model 6) as the virtual human response.
